# Comorbid Post-Traumatic Stress Disorder and Opioid Dependence

**DOI:** 10.7759/cureus.1647

**Published:** 2017-09-03

**Authors:** Rikinkumar S Patel, Ahmed Elmaadawi, Suhayl Nasr, John Haskin

**Affiliations:** 1 Department of Psychiatry, Northwell Zucker Hillside Hospital; 2 Psychiatry, Beacon Health System

**Keywords:** post-traumatic stress disorder, opioid dependence, ptsd, substance use, addiction

## Abstract

Post-traumatic stress disorder (PTSD) is predominant amongst individuals addicted to opioids and obscures the course of illness and the treatment outcome. We report the case of a patient with major depressive disorder and opioid dependence, who experienced post-traumatic stress disorder symptoms during a recent visit to the inpatient unit. The similarity of symptoms between post-traumatic stress disorder and opioid dependence is so high that, sometimes, it is a challenge to differentiate between these conditions. Since opioid withdrawal symptoms mimic hyper vigilance, this results in an exaggeration of the response of patients with post-traumatic stress disorder. This comorbidity is associated with worse health outcomes, as its pathophysiology involves a common neurobiological circuit. Opioid substitution therapy and psychotherapeutic medications in combination with evidence-based cognitive behavioral therapy devised for individuals with comorbid post-traumatic stress disorder and opioid dependence may improve treatment outcomes in this population. Therefore, we conclude that the screening for post-traumatic stress disorder in the opioid-abusing population is crucial. To understand the underlying mechanisms for this comorbidity and to improve the treatment response, further research should be encouraged.

## Introduction

Opioid addiction is the major culprit for the current ongoing epidemic, with around 20,101 overdose deaths due to prescription pain relievers, and 12,990 overdose deaths related to heroin in 2015 [1]. In the year 2012, opioids were prescribed to 259 million Americans, which is equivalent to giving every American adult a bottle of pills [2]. Women are more prone to be on prescription pain relievers as compared to men.

The estimated number of drug-related emergency department (ED) visits increased from 2.5 million to 4.6 million during 2004-2009, according to the substance abuse and mental health services administration (SAMHSA) drug abuse warning network (DAWN). Out of which, the highest numbers of ED visits were recorded for oxycodone products (242.2%) and hydrocodone products (124.5%), all of which had a statistically significant increase over the five-year period. The most common reported drugs in case of the nonmedical use category of ED visits were opiate/opioid analgesics, which was present in 50% of nonmedical-use ED visits and the most frequently reported opioids were among single-ingredient formulations (e.g., oxycodone) and combination forms (e.g., hydrocodone with acetaminophen) [3].

With that, the chances of having severe post-traumatic stress disorder (PTSD) symptoms were almost four times higher for persons with prescription opioid and sedative-use problems, followed by prescription opioid and cocaine use-related problems. Also, while examining the co-occurrence of prescription opioid use problems and PTSD, females also had higher prevalence rates than males. A moderate association between age (18–34-year range) and the co-occurrence of PTSD symptoms and prescription opioid use problems were found, as a result of which younger adults may be at increased risk [4]. Considering avoidance being a major feature of PTSD, the likelihood of patients’ under-reporting PTSD symptoms is high.

## Case presentation

Let’s take the case of our female patient who was a 30-year-old African American, transferred to the acute adult psychiatry unit from the main hospital, where she was diagnosed with severe unilateral eye pain, depressed mood, and suicidal ideation. Right from the first-day visit at the unit, she complained of being depressive, coupled with anxiety and recurrent suicidal ideation. She also had severe right-sided eye pain and demanded appropriate PRN (as needed). However, she did not suffer from any homicidal ideation or auditory or visual hallucinations on Day 1.

The patient was a casually dressed, well-groomed female of medium stature, average build, and average weight, shivering and covered with a blanket wrapped around her body and wearing sunglasses as to avoid direct bright light. During the interview on Day 2, she was very tearful and could not maintain direct eye contact. Her speech pattern was slow and sparse, her  mood was 'anxious' and ‘depressed.’ She appeared to be dysphoric and with a labile affect.

She was also going through hopelessness, a feeling of guilt, and low energy. Despite this, she balanced a long train of thought with no apparent disorganization and had a goal-directed process of reflection. Post her breakup with her boyfriend and past experience in prostitution, she appeared to be focused on these incidences. Her constant ruminations about these incidences stimulated her negatively and painfully. She wished for a normal life and had remorse.

Even with this, she denied any thoughts of hurting herself, while demonstrating no signs of aggression toward others. Hallucinations are one of the predominantly depressive characters. Our female patient reported hallucinations on Day 3, which was more during the nights and grew worse during the day. It started with a vision involving the dead body of her mother, then gradually became more complex and graphic over the past six months. She never reported this symptom during her previous psychiatry visits.

During her admission, she also reported auditory hallucinations, as she hears the voices of her ex-boyfriend and of multiple customers of her on a prostitution website. These voices were felt during the interview and told her to stop wasting time and get back to her work on the prostitution website. She reported nightmares of her mother’s demise, which disrupted her sleep.

Although she appeared to be reliable toward her self-disclosure, she displayed difficulty in describing her emotions and memories of the incident. One cannot be sure of her judgment and her insight was poor. She reported withdrawals from opioid use, such as racing thoughts, palpitations, lower back pain, and stomach cramps and craved for PRN pain medications even more.

In the last two months, she visited the ED several times for eye pain, back pain, agitation, and altered mental status; and was diagnosed with polysubstance abuse and opioid overdose. Post receiving the appropriate treatment, she was discharged with follow-up at outpatient psychiatry, but since then, the patient did not return as an outpatient.

Since her parents separated, our female patient lived in different foster homes, when she was 10 years of age. At the age of 12, she was traumatized due to sexual abuse by her father, who also forced her to take 750 mg hydrocodone daily for leisure. Things got even worse when she was in her 20s when her mother used to supply her 90 mg percocet every month. If one looks at her family history, this should not come as a shock when her father had opioid dependence too and her mother was diagnosed with bipolar disorder I and opioid dependence as well. Due to opioid overdose, the patient’s mother died in 2015, and in the last six months, she started having frequent visual hallucinations of her mother’s dead body and nightmares. Since her divorce and mother’s demises, she became homeless and had been staying with her boyfriend for about a year. The situation became worse when she was forced by her boyfriend to work for him on the prostitution website, and he used to provide her 200 mg fentanyl patches and morphine pills daily. Just three days prior to the current admission, the female patient was physically and emotionally assaulted by her boyfriend, as she resisted working as a prostitute on the prostitution website.

The female patient was involuntarily admitted to the acute adult psychiatric unit of the hospital for depression and suicidal ideation; however, by Day 3, she suffered a high level of distress as a result of her hallucinatory experiences and nightmares for which she needed urgent assessment, observation, and treatment. After that, a routine blood work and urine drug screen showed positive for opiates, amphetamine, cocaine, and tetrahydrocannabinol (THC).

Further, her cognitive functions (concentration, attention, information processing, executive functions) were intact and neurological examination was normal. The emotion was controlled with a tendency for avoidant behavior; however, there was complexity seen in processing and expressing emotions adaptively. Self-esteem was beaten down with feelings of inferiority and inefficiency. On Day 1, initial diagnosis on admission was ‘recurrent major depressive episode, opioid use disorder, cocaine use disorder, and amphetamine use disorder,’ and on Day 3, after further psychiatric evaluation, she was diagnosed with ‘post-traumatic stress disorder.'

Post that, she was placed on citalopram 10 mg once daily for depression and lorazepam 1 mg PRN for anxiety attacks on Day 1. On Day 3, she reported nightmares and auditory and visual hallucinations to the attending physician during the interview and for which she was diagnosed with post-traumatic stress disorder. Due to her severe nightmares, which resulted in disrupted sleep patterns along with graphic visual hallucinations and voices that were worsening and present even during daytime, she was given quetiapine 50 mg QHS (at bed time) and haloperidol 5 mg PRN to help ease with her psychotic symptoms. She started fentanyl patches 100 mg for opioid withdrawal symptoms. Within two days, quetiapine was gradually increased to 50 mg BID (twice daily) with no significant effect to the perceptual disturbances, but since the development of more severe nightmares, prazosin 1 mg QHS was added.

Because the morning quetiapine made her drowsy and tired, she preferred to stay in bed due to agony and insomnia. Due to quetiapine being ineffective in the psychotic symptomatology, medication was gradually titrated to quetiapine 300 mg QHS by Day 6. Also, Fentanyl patches were slowly tapered down to 25 mg and she no longer exhibited opioid withdrawal symptoms. She frequently complained of eye pain and asked for pain medication PRN on Day 8. She was diagnosed with corneal abrasion and given erythromycin ointment and tramadol 50 mg PRN. On the ninth day, she reported no signs of any nightmares or auditory or visual hallucinations and when asked to rate her depression and anxiety, she rated one out of 10. Her exit diagnosis was “post-traumatic stress disorder, opioid dependence disorder, and recurrent major depressive episode." 

## Discussion

The initial diagnosis of our female patient was detected as major depressive disorder and opioid dependence, but our impression of her is that she had used opioids in part to self-medicate symptoms related to her underlying undiagnosed PTSD. Furthermore, the patient reported that her PTSD symptoms persisted beyond the acute, subacute, and chronic withdrawal periods, which indicates that these symptoms represented a mere artefact of opioid withdrawal.

When the human body is exposed to trauma and stress, it causes corticotropin-releasing hormone (CRH) to stimulate an increased release of proopiomelanocortin (POMC), which is divided into the adrenocorticotropic hormone (ACTH) and beta-endorphin. While ACTH increases alertness and arousal, which leads to the fight-or-flight response, beta-endorphin reduces both emotional and physical pain [5]. Hippocampus, anterior cingulate cortex, and medial prefrontal cortex (mPFC) respond in a different manner in PTSD and PTSD with co-occurring opioid dependence. These brain regions show diminished activity in PTSD but augmented activity in PTSD with comorbid opioid dependence in response to exposure to traumatic events [6]. This explains why PTSD patients use opioids to recover their symptoms, namely, depression, anxiety, insomnia, and other mood issues [7].

Cortisol and norepinephrine are considered to be two neurochemical systems that are critical in the case of the stress response. CRH triggers the hypothalamic pituitary adrenal axis (HPA axis) and acts centrally to mediate fear-related behaviors, thereby triggering other neurochemical responses to stress such as the noradrenergic system via the brainstem locus coeruleus [8]. Noradrenergic overstimulation in individuals with PTSD may also increase their predisposition to illicit drug use for self-medication, especially for substances with sedating properties, such as alcohol, opioids, and benzodiazepines [9]. Opioid withdrawal also stimulates the noradrenergic system in the locus coeruleus, thereby adding to the increased predisposition to chronic opioid use to prevent symptoms of PTSD and opioid withdrawal in this population [10].

## Conclusions

Because of the significant rates of comorbid psychiatric and substance-use disorders, cases with opioid-use disorders should be proactively screened and assessed for PTSD and those with PTSD should be proactively screened and evaluated for opioid-use disorders. If an individual is diagnosed with comorbid psychiatric and substance-use disorders, specific treatment should be initiated for both disorders and should occur concurrently using coordinated, evidence-based treatments, including effective psychosocial interventions and pharmacotherapies. This shows that complex cases of opioid dependence with comorbid PTSD, that is, presenting with severe nightmares and hallucinatory experiences can be successfully treated with pharmacotherapy and supportive psychotherapy to understand an individual’s functioning and character dynamics. A more biopsychosocial approach is crucial in integrating all aspects of the patients' history in a meaningful way to provide adequate psychiatric care.

## References

[1] Rudd RA, Seth P, David F, Scholl L (2016). Increases in drug and opioid-involved overdose deaths - United States, 2010-2015. MMWR.

[2] Opioid Painkiller Prescribing (2017). Opioid Painkiller Prescribing. 2014, Centers for Disease Control and.

[3] SAMSHA SAMSHA (2017). Drug-Related Hospital Emergency Room Visits. https://www.drugabuse.gov/publications/drugfacts/drug-related-hospital-emergency-room-visits.

[4] Meier A, Lambert-Harris C, Mcgovern MP, Xie H, An M, Mcleman B (2014). Co-occurring prescription opioid use problems and posttraumatic stress disorder symptom severity. Am J Drug Alcohol Abuse.

[5] Volpicelli J, Balaraman G, Hahn J, Wallace H, Bux D (1999). The role of uncontrollable trauma in the development of PTSD and alcohol addiction. Alcohol Res Health.

[6] Sell LA, Morris JS, Bearn J, Frackowiak RS, Friston KJ, Dolan RJ (2000). Neural responses associated with cue evoked emotional states and heroin in opiate addicts. Drug Alcohol Depend.

[7] Holbrook TL, Galarneau MR, Dye JL, Quinn K, Dougherty AL (2010). Morphine use after combat injury in Iraq and posttraumatic stress disorder. N Engl J Med.

[8] Arborelius L, Owens MJ, Plotsky PM, Nemeroff CB (1999). The role of corticotropin releasing factor in depression and anxiety disorders. J Endocrinol.

[9] Bremner JD, Southwick SM, Darnell A, Charney DS (1996). Chronic PTSD in Vietnam combat veterans: course of illness and substance abuse. Am J Psychiatry.

[10] Parlato R, Cruz H, Otto C (2010). Effects of the cell type-specific ablation of the cAMP-responsive transcription factor in noradrenergic neurons on locus coeruleus firing and withdrawal behavior after chronic exposure to morphine. J Neurochem.

